# The Fidget Factor and the obesity paradox. How small movements have big impact

**DOI:** 10.3389/fspor.2023.1122938

**Published:** 2023-04-03

**Authors:** James A. Levine

**Affiliations:** Fondation Ipsen, Fondation de France, Paris, France

**Keywords:** physical activity, fidget, obesity, diabetes, neural circuits, school–environment relations

## Abstract

The hypothesis is that the Fidget Factor is the innate neurological pulse that propels humans and other species to move to support their health. Fidgets, previously thought to be spontaneous, are neurologically regulated and highly ordered (non-random). Modern societies being chair-based overwhelm Fidget Factor pulses and consequently inflict chair-based living for transportation, labor, and leisure. Despite impulses firing through the nervous system, people sit because environmental design overwhelms the biology. Urbanization and chair-based societies were designed after the industrial revolution to promote productivity; however, the consequence has been opposite. Crushing the natural urge to move—the Fidget Factor—is a public health calamity. Excess sitting is associated with a myriad of detrimental health consequences and impairs productivity. Fidgeting may reduce all-cause mortality associated with excessive sitting. The Fidget Factor offers hope; data demonstrate that workplaces and schools can be designed to promote activity and free people's Fidget Factors. Evidence shows that people are happier, healthier, wealthier, and more successful if their Fidget Factors are freed.

## Introduction

The hypothesis is that the Fidget Factor is an innate and healthful drive for human beings to move. It has long been assumed that the small movements people make are spontaneous and random. The hypothesis addressed in this paper is that these small movements, called fidgets, are neurologically regulated, programmed, and highly ordered. Fidgets trigger body and limb movements and locomotion. While in agricultural times this drive was unfettered, in postindustrial modern environments, chair-based cues are pervasive and suppress people's innate drives to move. Because of suppressed Fidget Factors, people sit excessively. Excess sitting is associated with physical and mental illness and premature death. Solutions exist to reverse sedentariness and allow people's natural Fidget Factors to propagate healthful movement.

## Fidget

Meriam-Webster (1) defines the verb “to fidget” as “to move or act nervously or restlessly” and the noun “fidget” as a “nervous movement.” Fidgets characterize the spectrum of species that range from nematodes to humans. Common to all understandings of fidgets is that they are nervous in origin.

## Neurological regulation of the Fidget Factor

The Fidget Factor is under neurological regulation and integrated in the human energy regulation cycle (2–13). According to one example, Orexin A is a neuropeptide that is produced in caudal hypothalamic regions and projects throughout the neuraxis where it enhances arousal and stimulates the Fidget Factor (4). Orexin is one of several mediators of the Fidget Factor. When orexin is injected into the paraventricular nucleus in rats, it precipitates fidgets in a dose-dependent fashion (14). Paraventricular nucleus injections of an orexin receptor antagonist are associated with decreases in Fidget Factor responses (14). In transgenic mice where orexin-containing neurons are ablated, the phenotype includes inactivity and late-onset obesity, despite the transgenics eating less than non-transgenic littermates (15). Orexin A also stimulates the Fidget Factor codependently with feeding behavior (16) when injected into the lateral hypothalamus. Orexin impacts several hypothalamic nuclei to regulate fidgeting.

Orexin is not the only central mediator of the Fidget Factor; other neuromodulators include cholecystokinin, agouti-related protein, corticotropin-releasing factor, neuromedin U, neuropeptide Y, leptin, the serotonergic system, and ghrelin (12, 13, 17, 18). Several brain loci are involved as well; *nucleus accumbens*, for example, is considered the neural interface between motivation and movement and controls fidget-like movements (19). Movement is important in multiple facets of life such as feeding, foraging, and fleeing, and so, it is not surprising that the Fidget Factor represents the neurological end product of several central control centers and circuits.

It is not only mammals that have Fidget Factors. Molecular studies in zebrafish show that orexin mediates swimming activity and energy balance (20); worms fidget too (21). The Fidget Factor is ubiquitous in zoology and under intricate neurological control (22).

### Are fidgets random?

**Figure 1:**
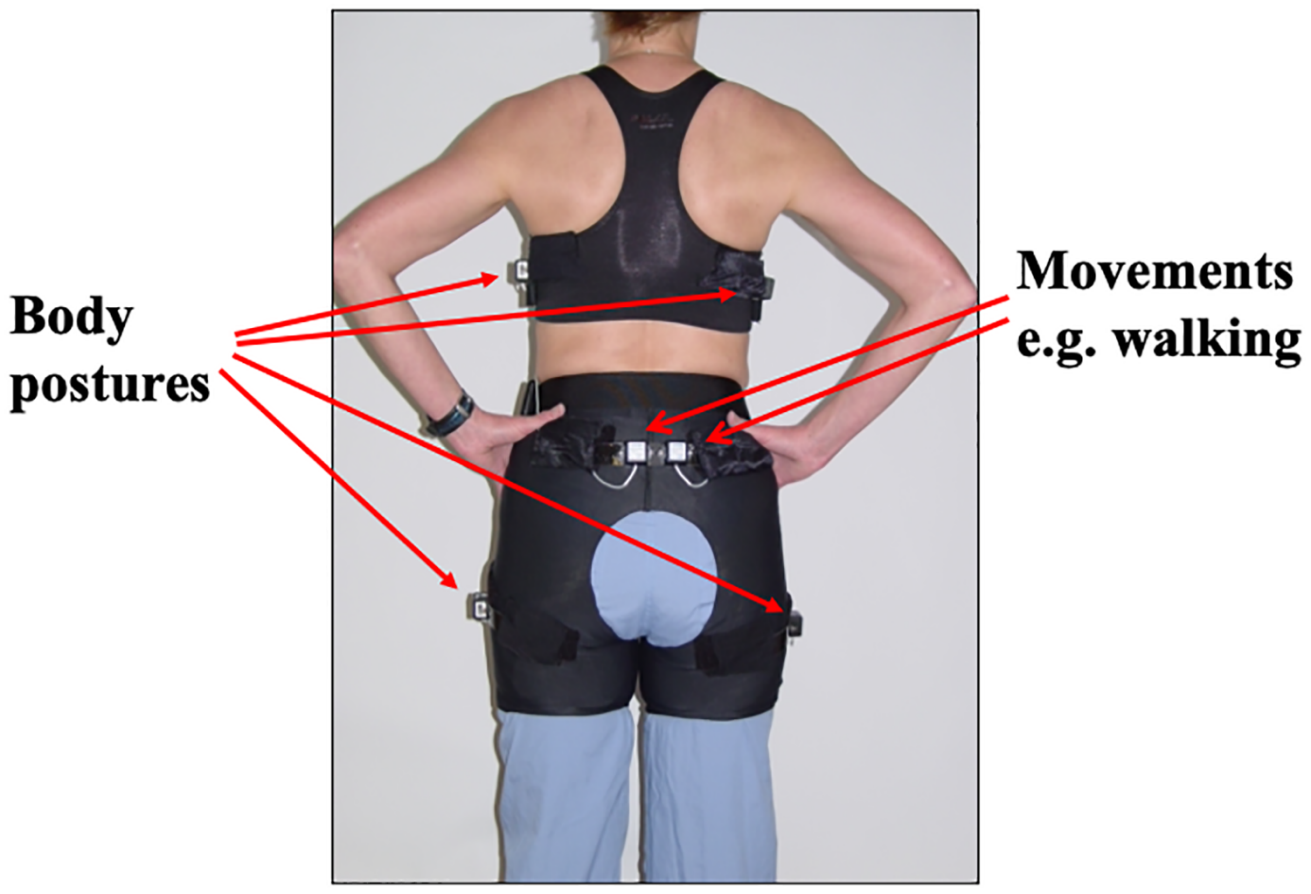
Multiple inclinometers and accelerometers integrated into underwear. Reproduced from Levine et al. (23).

It has long been assumed that fidgets are spontaneous and random. To examine movements in free-living people, underwear was designed that included multiple sensors embedded in upper and lower undergarments (23); the underwear (Figure 1), thereby, captured all free-living body postures and body movements over 13 axes of motion every half second. The underwear sensing system was used to examine 10,362 free-living walking events and day-time and nighttime postures and movements in 21 people (24). Free-living walking comprised many (−47) short-duration (<15 min), low-velocity (−1 mph) walking bouts. Importantly, there was remarkable within-person consistency for the subvariables of free-living daily activity such as the number of walks a person takes per day, free-living walking velocity, and overall walking time (*r*^2^ values ranged 0.6–0.8). This suggests that free-living movement is not random and therefore regulated.

Using modern mathematics, human fidgeting can be analyzed for entropy, which is a measure of randomness (25–30). ApEn determinations of entropy confirm that fidget-like human movements are non-random in infants (27, 29), young adults (25), adults, and in the aged (28, 31). When the orderliness of human movement is disrupted (e.g., jumping back in response to a fast-moving car), a person's physiological disorderliness with respect to movement rapidly self-corrects (32). There is the fitness effect too; the more physically conditioned a person the more orderly their fidgets (33). Human fidgets are highly ordered. The programed orderliness of human movement can be pathologically disrupted by illnesses such as in Parkinson's disease (34, 35) or by direct manipulation, for example, by asking women to walk in heels vs. flat shoes (30). Mechanistically, the rhythmicity of the Fidget Factor is mechanistically linked to the Clock gene, which is central to circadian timing; homozygous Clock mutant mice exhibit temporally disrupted activity patterns (36). The Fidget Factor is, therefore, organized and mechanistically encoded most likely *via* central modulators. Human movements are neither random nor spontaneous; as George Gershwin wrote, “I got rhythm” (Treasure Girl 1928). People have more rhythm than they realize. The Fidget Factor is an outward manifestation of an inner rhythm to move.

Noting the above and the relevance of fidgets in multiple species and across several genera, we can better define “a fidget” in a biological context. The initial definition of a fidget discussed above was “a nervous movement.” A fidget might be better defined as “a neurologically programmed rhythmic movement of a body part.” Under normal functioning, a fidget might be the spark that predicates a larger orchestrated movement whereby a foot flinch fidget begets a leg extension and precipitates a walk. Under pathological conditions, such as mutation of the HTT gene, a fidget might extend into the choreoathetoid movements that characterize Huntington's Chorea (37).

**Figure 2:**
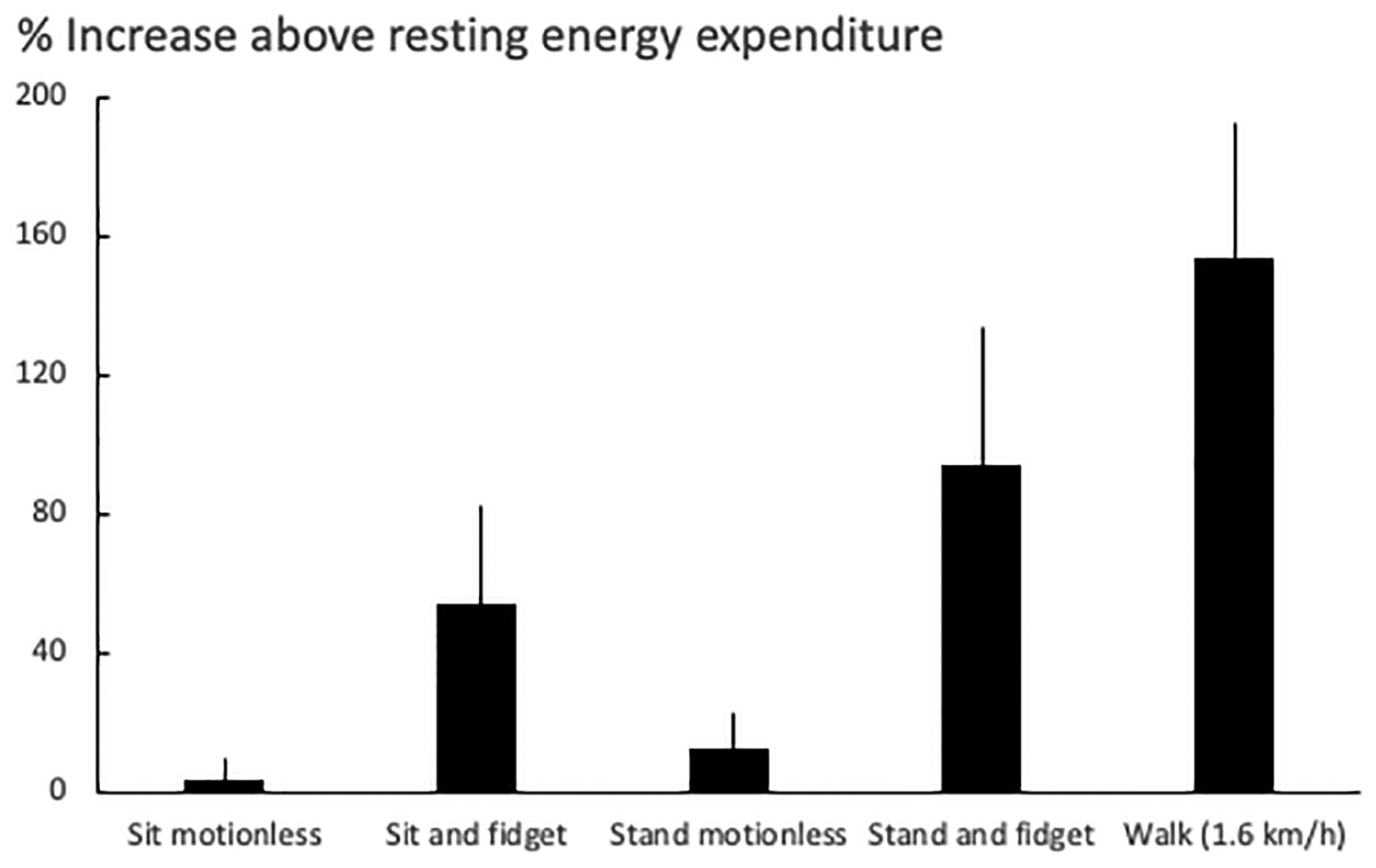
The effect of fidgeting in different postures on human energy expenditure. Data (mean ± SD) are taken from 24 subjects (17 women and 7 men; body weight: 76 ± 21 kg) (38).

There are little fidgets (e.g., the tap of a finger) and big fidgets (e.g., the crossing of legs). Both fidgets have numerically different effects on human physiology (Figure 2).

## Physiological relevance of the Fidget Factor

The Fidget Factor is centrally regulated and ordered, however, if it is not physiologically meaningful, its significance is less. The impact of the Fidget Factor on human physiology was examined in 16 lean volunteers (23). The subjects ate all their meals at a research center for 10 weeks; all meals were chemically analyzed for caloric content. For the first 2 weeks, each person was fed to establish the calorie intake necessary for weight maintenance to determine how many calories each volunteer required for achieving a steady state. Thereafter, each volunteer was overfed by 1,000 additional kcal per day, i.e., if a subject ate 2,700 kcal/day to maintain a steady state, this was increased to 3,700 kcal/day. In this fashion, each volunteer received an excess 56,000 kcal over 8 weeks.

**Figure 3:**
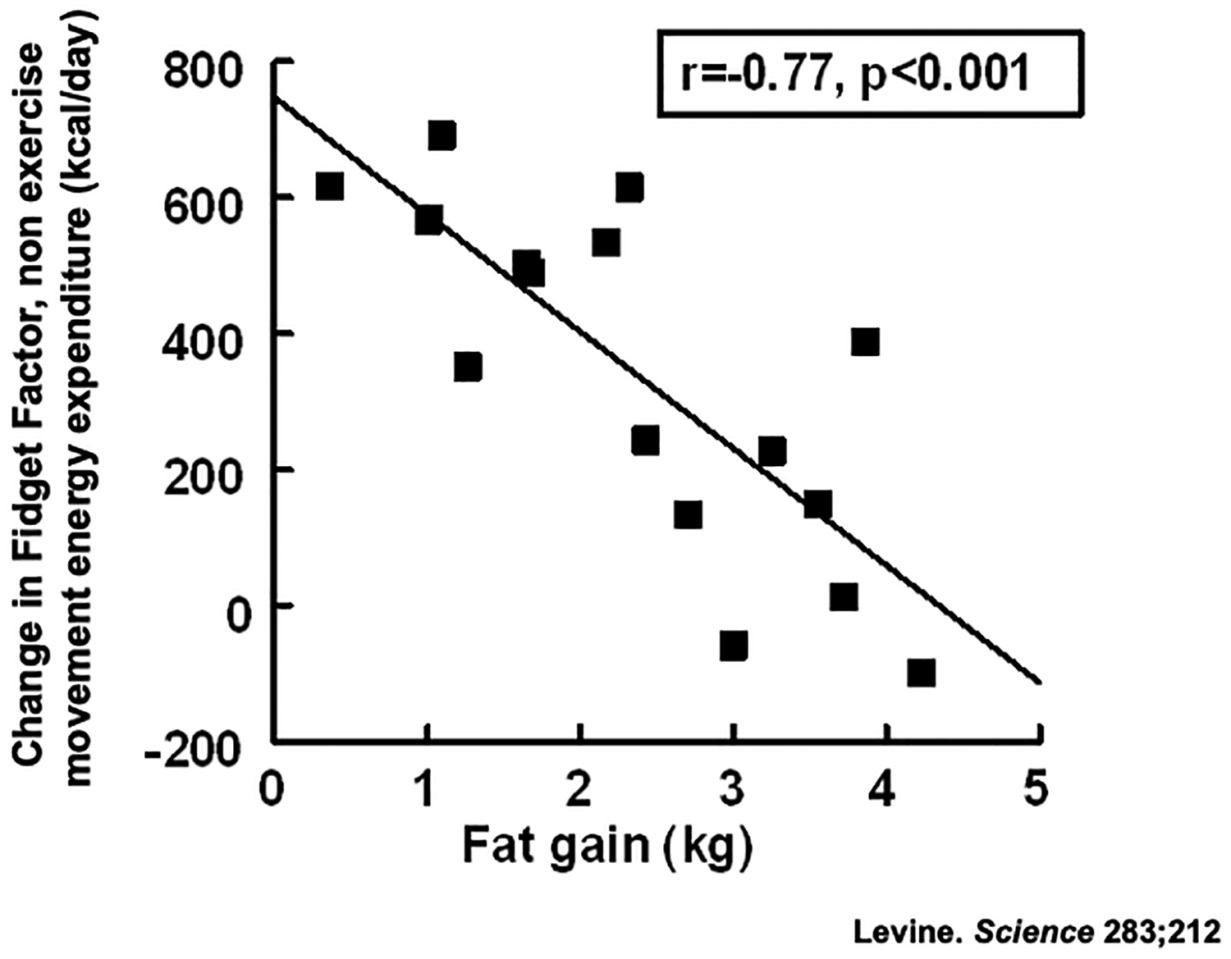
Activation of the Fidget Factor, non-exercise activity thermogenesis (NEAT) in 16 individuals overfed by 1,000 kcal/day for 8 weeks. Body fat was measured using validated dual x-ray absorptiometry (23).

There was a 7-fold variation in people's susceptibilities to weight gain (Figure 3). Some individuals were remarkably resistant to fat gain with overfeeding because they activated their Fidget Factors; the energy expended through non-exercise movement (39, 40). Increased non-exercise movements with overfeeding expended up to 700 kcal/day above usual energy expenditure. A statistically significant negative correlation (Figure 3) between fat gain and non-exercise movements suggested a mechanistic link, a proposition supported by animal data (41). The Fidget Factor is central in energy homeostasis.

Overfeeding can increase energy expenditure *via* the Fidget Factor by 700 kcal/day. How is this possible without one going to a gym? Further overfeeding experiments were conducted but with subjects wearing multisensor undergarments (Figure 1). The results (42) showed that people activate their Fidget Factors by increasing locomotion. This is not achieved by exercise but by subconsciously and imperceptibly adjusting the mechanics and energetics of walking.

If overfeeding is important in a person's susceptibility to fat gain, is the Fidget Factor important in obesity? To understand the role of the Fidget Factor in mild obesity, lean and obese office workers were compared using the multisensor system shown in Figure 1. The results were dramatic. Lean people have activated Fidget Factors; they stand and move 2 1/4 h per day more than people with obesity. This appears to reflect a biological predisposition, because people who are active at work are active during their leisure time and people most sedentary at work are those most sedentary at home (24, 42). All subjects in these studies lived in obesogenic chair-based environments. Those with activated Fidget Factors were thin. For others, pervasive environmental cues to sit overrode the physiological impulse to move, resulting in obesity.

If the Fidget Factor changes in response to overfeeding and with obesity, how is it influenced by starvation and weight loss? Regardless of species, Fidget Factors change with acute starvation in a predictable fashion (7, 20, 43). Initially, starvation increases Fidget Factor activities, which is ascribable to foraging behavior. If food remains unavailable, physical activity will then progressively decline. When chronic caloric restriction results in weight loss, people's Fidget Factors reset to a new norm (44–46). Training and fitness levels also impact Fidget Factors in humans (47) and in other species (48–51). The Fidget Factor is clearly central in energy homeostasis.

The Fidget Factor is modulated across the life span (52). *In utero* (53), “spontaneous” movements are associated with development and limb growth (53). A systematic review of 15 studies showed a significant relationship between the quality of fidgety movements at 8–20 weeks post term and the infants' neurodevelopmental outcome. This association is specific as another systematic review showed, “the presence of abnormalities in the quality of fidgety movements at 12 weeks adjusted age is more predictive of adverse outcomes than abnormal writhing movements” (54). Fidgets in an infant are important for learning to walk and cognitive development (55, 56). Children are more fidgety than adults (48, 57), and then, the Fidget Factor declines with aging (58, 59) which may be important in sarcopenia (60). This pattern of high activity into adulthood and the decline with ageing is mirrored in other mammals (61), flies (62), and worms (21).

Fidgeting is not limited to humans. Multiple behaviors in non-human primates in the wild resemble human fidgeting (63). In primate experiments, cortical activity regulated cytoskeletal-associated protein and brain-derived neurotrophic factor expressions are strongly correlated with fidgeting activity (64). Furthermore, in monkey models of human neurological disease, phenotypes with diminished fidget-like movements mirror the human condition (65). Fidget-like movements occur across genera and are moderated over the life span in fish, flies, and worms (20, 21, 62). Interestingly, worms show a similar diminishment in “fidgets” (spontaneous movements) over the life span compared with humans, becoming near motionless just prior to death.

The Fidget Factor, controlled by multiple neurological loci, has an important role across the life span of many species that are central for growth and energy homeostasis.

## Environmental impact on the Fidget Factor

There is an intersect between environment and biology. The Fidget Factors of some people appear to be insufficient to override obesogenic chair-based environments; such individuals sit too much and develop obesity. People whose Fidget Factors override environmental cues to sit remain mobile, active, and thin. How quantitatively important are these environmental drives on the Fidget Factor?

Since the Industrial Revolution, most of the world's population has relocated from agricultural communities to urban centers (66–69). Urbanization was initially meant to support factory production, but this developed into chair-based offices. To what extent has this massive demographic shift impacted the Fidget Factor?

Movement sensing undergarments (Figure 1) were used to compare agricultural and urban populations in Jamaica (70). The agricultural community included field workers, teachers, dancers, hairdressers, and educators. They were compared with weight-matched controls working in offices in the capital, Kingston. Ambulation was 60% greater in agricultural Jamaicans than in urban dwellers. Agricultural Jamaicans sat 4 h/day less than Americans with obesity (336 ± 68 min/day vs. 562 ± 78; *P* < 0.001). This illustrates the amount of excess sitting that resulted in response to urbanization—4 h more. Genetic vulnerability may help explain why some people respond more to environmental cues to sit than others (71). Nonetheless, environment is a key driver of the Fidget Factor. If people were liberated from their chairs, they could move for 4 h more every day (72, 73).

Agricultural workers sit between bouts of physical work and for leisure (73); the default posture is to work standing and exothermically and walk for transportation. In modern chair-based societies, sitting is the default posture and cars are used for transportation. For many people who live in chair-based societies, their Fidget Factors are suppressed; if these are released, people can healthily tolerate 4 h less sitting per day.

In contrast, there is ample empirical evidence from the affective computing literature that knowledge workers engage in extensive hand fidgeting during long and intense bouts of cognitive work (74). Furthermore, when the fidgeting cycle is disrupted during pathological conditions such as Huntington's Chorea (37), there is cognitive decline. It is fascinating how the state of anxiety is linked to fidgeting; in extreme anxiety, under adrenergic drive, tremulousness and elevated energy expenditure occur in concert (75). Environments impact biology: fidgetiness is a marker of heightened anxiety.

In a similar way that environment can impact a person's inmate fidgetiness, so does culture (76–79). Cultural group differences influence a person's likelihood to fidget; people from different cultures fidget differently in response to anxiety (77) and even when deliberately deceiving others (76).

While people have innate neurological drives to fidget and move, environment and culture can quash these drives, but at what cost?

## Health implications of the Fidget Factor

Chair-based living and the environmental cues associated with it override peoples' natural tendencies to move—their Fidget factors are suppressed (80). This would not be problematic except that excessive sitting is harmful to health (81–83). Excess sitting is associated with metabolic, musculoskeletal problems, malignancy, and mortality (84–88); examples include cardiovascular disease, obesity (89), type two diabetes (90), hyperlipidemia, cardiovascular disease (91), hypertension, lower back pain (92), carpal tunnel syndrome, venous stasis (93), low mood, and a greater risk of malignancy (94–99).

Why is excess sitting so harmful? According to one example (100), healthy volunteers attended a research center and carried out normal chair-based work and leisure activities. Blood glucose was monitored continuously throughout the experiment. Breakfast, lunch, and dinner were provided and meal-related changes in blood glucose were recorded after each meal. For these subjects, their meal-associated glucose responses (incremental glucose area under the curve) were 9.6 mmol/L/270 min. The same volunteers repeated the same chair-based protocol with the duplicate meals except that after each meal, the subjects took a 30-min stroll at 2 km/h. Their meal-associated glucose responses were halved (4.5 mmol/L/270 min; *P* = 0.002), a finding replicated by others (101–103). Noting that meal-associated glucose responses predict the development of type 2 diabetes (104, 105), this experiment helps explain why chair-suppressed Fidget Factors are associated with type 2 diabetes (90, 106).

Similar experiments show the harmful effect of sedentariness on lipid metabolism (88) and that slow walking raises lipoprotein lipase activity beneficially by approximately 8-fold (107, 108). Furthermore, sedentariness is associated with proinflammatory markers, depressed sympathetic activity (109), and elevated insulin-like growth factors (110), which, in turn, are associated with malignancy (111). It is interesting to note from Morishima's carefully conducted studies of bilateral popliteal artery flow–mediated dilation that prolonged sitting-induced leg endothelial dysfunction may be prevented by fidgeting (112).

Anatomic and ergonomic analyses explain why musculoskeletal problems such as back pain and other mechanical disorders are linked to excess sitting (113, 114). Prolonged sitting is associated with spine flexion, low back disorders, disc stress, and pain (115). Back muscles are activated when a person stands, and similarly, the trunk muscles represent a counterbalance (116, 117). The musculoskeletal system is hurt when a person sits for hours without break.

It is not well understood as to why cognitive skills and mental health issues, especially depressed mood, are linked to excess sitting (118), but they are. Multiple prospective studies show that walking helps in preventing depression (119). An active body begets a happy mind!

Excess sitting has substantial detrimental health consequences and is associated with at least 34 different chronic diseases and conditions (113). The Fidget Factor is a pulsatile neurological impulse to move. Millions of people are sedentary because their natural Fidget Factors are suppressed by chair-based environments.

Almost no fidgeting intervention studied exist, epidemiological data suggest that fidgeting is associated with lower mortality risk (120). Analyzing data from the UK Women's Cohort Study, Hagger-Johnson et al. conclude, “Fidgeting may reduce the risk of all-cause mortality associated with excessive sitting time” (85).

The health consequences of sedentary living are calamitous, and people perish prematurely. If people were able to respond naturally to their innate Fidget Factors and move more, would they be healthier?

## Fidget Factor therapy

Scalable studies have taken place in workplaces and schools and demonstrate that Fidget Factor–permissive environments can be designed to reverse sedentariness and enable people to move (121). Such interventions are not straightforward because chair-based cues are pervasive at work and during leisure (122). Consequently, environmental redesign is only one part of the solution. Behavioral change strategies (123) are critical (Table 1) to help people reverse sedentariness, move (124), and “liberate” their Fidget Factors.

**Table 1:** Evidence-based behavioral change techniques that are effective for improving physical activity in healthy sedentary adults (124).

Physical activity intervention effectiveness (behavior change)	Effectiveness at follow-up: (behavior change maintenance)
Biofeedback	Action planning
Demonstration of the behavior	Instruction on how to perform the behavior long term
Behavior practice/rehearsal	Prompts/environmental cues
Graded tasks	Behavior practice/rehearsal
	Graded tasks
	Self-reward

Workplaces and schools impose a group dynamic that is important in supporting individual change. Group behaviors are well studied in animals (125) but less in humans (126, 127). Although group behavioral dynamics are not well understood, workplace productivity is evaluated using group-based criteria such as a company's profitability. Similarly, schools are compared against whole-school performance criteria. Fidget Factor–permissive environments facilitate a healthier group dynamic, physically, mentally, and productivity wise.

The economic value of Fidget Factor–permissive environments has been established. Returns on investments have been measured in workplace and school interventions and have been found to be positive (128–131) and associated with improved health behaviors, decreased absenteeism, and better mental health (132–134). Importantly, employee wellbeing has become included in a company's Environmental, Social, and Governance (ESG) metrics, which are the standards socially conscious investors apply to screen investments. Workplace wellbeing measures are likely to expand.

Taken *en masse*, workplace interventions are associated with improved physical and mental health (135) and improved employee wellbeing and productivity (86, 136–141). Similarly in school children, active learning at the expense of chair-based learning is associated with improved education and health (142–145). These programs pay for themselves (146, 147) and the costs reflect the sum of healthcare cost savings, decreased absenteeism, and improved productivity/education. People are happier, healthier, wealthier, and more successful if their Fidget Factors are freed.

## Discussion

Fidget Factor, the rhythmic impulse to move, is programmed from deep within the brain stem. These impulses precipitate movements that range from barely perceptible fidgets to larger motions. Modern chair-based societies override people's innate Fidget Factors; people living in agricultural societies move 4 h a day more than overweight people in modern offices, suggesting that people who are not restricted by chairs naturally move several hours per day more than chair-based urban office workers (68, 69). Consequently, when people's Fidget Factors are suppressed, excess sitting is pervasive, and the physical and mental health consequences are dire. People die early from sedentariness. Fidget Factor–permissive environments enable people to be healthier, happier, smarter, and more productive (148–151). Solutions exist, but they need to be deployed.
